# HCTG-Net: A Hybrid CNN–Transformer Network with Gated Fusion for Automatic ECG Arrhythmia Diagnosis

**DOI:** 10.3390/bioengineering12111268

**Published:** 2025-11-19

**Authors:** Ni Xiong, Zibo Wei, Xuehua Wang, Yan Wang, Zhaohui Wang

**Affiliations:** 1 Department of Cardiology, Union Hospital, Tongji Medical College, Huazhong University of Science and Technology, Wuhan 430022, China; 2 Hubei Key Laboratory of Biological Targeted Therapy, Union Hospital, Tongji Medical College, Huazhong University of Science and Technology, Wuhan 430022, China; 3 Hubei Provincial Engineering Research Center of Immunological Diagnosis and Therapy for Cardiovascular Diseases, Union Hospital, Tongji Medical College, Huazhong University of Science and Technology, Wuhan 430022, China; 4 MOE Key Laboratory for Biomedical Photonics, Wuhan National Laboratory for Optoelectronics, Huazhong University of Science and Technology, Wuhan 430074, China; 5 College of Life Science and Technology, Huazhong University of Science and Technology, Wuhan 430074, China

**Keywords:** ECG arrhythmia detection, hybrid CNN–Transformer, gated fusion, deep learning, biomedical signal processing, cardiovascular diagnosis

## Abstract

Accurate detection of cardiac arrhythmias from electrocardiogram (ECG) signals is essential for the early diagnosis of cardiovascular diseases but remains challenging due to the complex, non-linear nature of ECG waveforms. This study proposes HCTG-Net, a Hybrid CNN–Transformer Network with Gated Fusion, designed to jointly capture local morphological features and long-range temporal dependencies in ECG data. The model employs a dual-branch architecture, where a residual CNN extracts localized waveform patterns and a Transformer branch models global temporal context. A learnable gated fusion mechanism adaptively balances and integrates features from both branches at the per-dimension level. Experiments conducted on the MIT-BIH Arrhythmia Database demonstrate that HCTG-Net achieves superior performance compared with existing methods, reaching an overall accuracy of 0.9946 and F1-score of 0.9711. Visualization results show well-clustered feature distributions, confirming robust feature learning, while ablation studies verify the complementary roles of the CNN, Transformer, and fusion modules. Overall, HCTG-Net offers a powerful and adaptive framework for automatic ECG-based arrhythmia diagnosis and holds strong potential for real-time clinical and wearable healthcare applications.

## 1. Introduction

Cardiovascular diseases (CVDs) remain the leading cause of mortality worldwide, accounting for nearly 17.9 million deaths each year according to the World Health Organization. Arrhythmias, representing abnormal heart rhythms, are among the most prevalent manifestations of CVDs and significantly increase the risk of stroke [1], sudden cardiac arrest [2], and heart failure [3]. The electrocardiogram (ECG) is a non-invasive, low-cost, and widely adopted diagnostic tool that records the heart’s electrical activity and plays a crucial role in detecting arrhythmias [4], myocardial ischemia [5], and conduction abnormalities [6]. Consequently, accurate and timely arrhythmia diagnosis from ECG signals is critical for improving clinical outcomes.

However, ECG interpretation remains a complex task due to the signal’s non-linear nature [7]. ECG waveforms contain morphological components such as P waves, QRS complexes, and T waves that occur across multiple temporal scales [8]. Clinicians identify arrhythmias mainly through inspection of beat-to-beat timing irregularities, especially variations in R–R intervals. Classical signal-processing techniques such as spectral analysis, autoregressive modeling, and time–frequency representations have also been employed to detect rhythm-related abnormalities and uncover physiological oscillations that may not be visible in the raw waveform [9]. These methods offer valuable insights but may not fully capture the complex morphological deformations and multi-scale temporal dynamics that arise during arrhythmic episodes [9]. Furthermore, ECG morphology is influenced by physiological variations, electrode placement, and noise sources such as baseline drift and electromyographic interference [10,11,12]. During arrhythmic episodes, substantial morphological and temporal variability further complicates automated feature extraction and classification [13]. These challenges make automated ECG arrhythmia detection both technically demanding and clinically important.

Early approaches relied on handcrafted feature extraction combined with traditional machine learning algorithms such as support vector machines (SVMs), k-nearest neighbors (k-NN), and hidden Markov models (HMMs) [14,15,16]. While these methods achieved moderate success in specific tasks, their dependence on expert-designed features limits scalability, generalization, and adaptability to diverse patient populations. In contrast, deep learning has transformed ECG analysis by enabling end-to-end feature learning directly from raw signals. Convolutional neural networks (CNNs) effectively capture local morphological patterns of ECG beats, while recurrent neural networks (RNNs) and long short-term memory (LSTM) architectures model sequential dependencies [17,18,19]. More recently, Transformer architectures, initially developed for natural language processing, have demonstrated strong potential for modeling long-range dependencies in biomedical signals [20,21,22]. Nevertheless, CNNs often struggle to encode global context, whereas Transformers may overlook fine-grained morphological details that are crucial for precise arrhythmia classification [23,24].

To address these limitations, hybrid CNN–Transformer architectures have gained increasing attention. By integrating CNNs’ local feature extraction capabilities with Transformers’ global contextual modeling, hybrid frameworks provide complementary advantages. However, most existing methods employ serial or cascaded designs, where the Transformer is stacked on top of CNN features [25,26,27]. Such designs may fuse information at a coarse level and fail to adaptively balance local and global contributions. Consequently, their feature representations can be suboptimal for complex arrhythmia patterns requiring both detailed morphology and long-range temporal understanding.

In this study, we propose a Hybrid CNN–Transformer Network with Gated Fusion (HCTG-Net), for automatic ECG arrhythmia diagnosis. HCTG-Net adopts a dual-branch architecture consisting of (1) a residual CNN branch for efficient extraction of local morphological features, and (2) a Transformer branch with positional encoding for capturing long-range temporal dependencies. A learnable gating fusion module dynamically aligns and balances the outputs of both branches on a per-dimension basis, ensuring adaptive integration of local and global representations.

The main contributions of this study are summarized as follows:

1. We propose HCTG-Net, a hybrid deep learning architecture that jointly models fine-grained local morphological features and long-range temporal dependencies from ECG signals.
2. We develop a gated fusion mechanism that adaptively aligns and balances CNN and Transformer features at a per-dimension level, enhancing representational complementarity.
3. We conduct comprehensive experiments on benchmark arrhythmia datasets, demonstrating that HCTG-Net achieves superior accuracy, robustness, and generalization compared to existing methods.

The remainder of this paper is structured as follows. Section 2 describes the dataset, preprocessing procedures, and the proposed HCTG-Net architecture. Section 3 presents the experimental setup, performance evaluation, and comparative results. Section 4 provides a discussion of the findings and their implications. Finally, Section 5 concludes the paper and outlines directions for future research.

## 2. Methods

**Figure 1:**
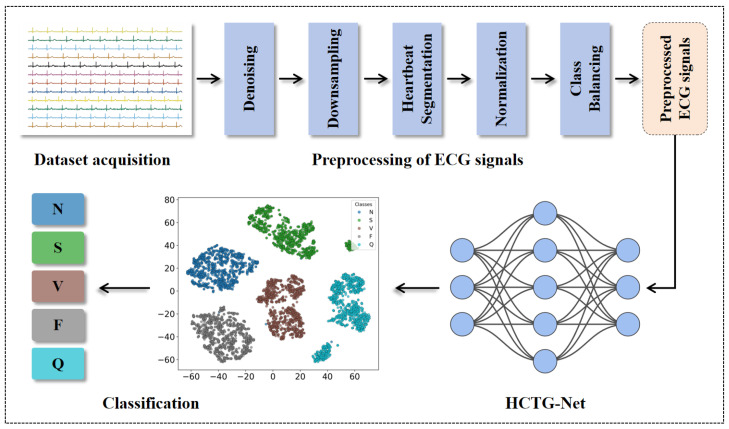
Overall workflow of the proposed HCTG-Net framework for automatic ECG arrhythmia diagnosis. N includes normal beats, left and right bundle branch blocks, atrial escape beats, and nodal escape beats. S includes atrial premature beats, aberrant atrial premature beats, nodal premature beats, and supraventricular premature beats. V includes premature ventricular contractions and ventricular escape beats. F represents fusion of normal and ventricular beats. Q corresponds to unclassified or unknown beats.

The proposed HCTG-Net framework for automatic ECG arrhythmia diagnosis consists of three main stages: dataset acquisition, preprocessing of ECG signals, and classification using a hybrid CNN–Transformer gated fusion network. The overall workflow is summarized in Figure 1.

### 2.1. Dataset

This study used the MIT-BIH Arrhythmia Database, which is one of the most widely adopted and influential benchmarks in arrhythmia research [28]. The database contains 48 half-hour two-channel ECG recordings collected from 47 subjects consisting of 25 men aged between 32 and 89 years and 22 women aged between 23 and 89 years at a sampling rate of 360 Hz. Each record includes two simultaneously acquired ECG signals. In most recordings, the upper signal corresponds to a modified limb lead II (MLII) collected from chest-mounted electrodes, while the lower signal typically corresponds to a modified precordial lead such as V1, occasionally V2, V4, or V5. This lead configuration was standard practice in the BIH Arrhythmia Laboratory to ensure robust detection of arrhythmic events. In all of our experiments, we have used ECG lead II as the input. Each record also includes expert annotations of beat locations and arrhythmia types, resulting in approximately 110,000 labeled heartbeats. Furthermore, the recordings contain realistic noise and artifacts such as baseline drift, muscle interference, and electrode motion. These noisy segments closely resemble real-world ECG signals encountered in clinical practice and wearable monitoring, making MIT-BIH an appropriate dataset for assessing the practical applicability of deep learning models. Detailed information about the MIT-BIH database is provided on PhysioNet at https://physionet.org/content/mitdb/1.0.0/ (accessed on 16 August 2025).

To ensure comparability with prior work, beat annotations were merged into five categories according to the AAMI standard [29]. Specifically, category N includes normal beats, left and right bundle branch blocks, atrial escape beats, and nodal escape beats, which share stable and characteristic morphology. Category S includes atrial premature beats, aberrant atrial premature beats, nodal premature beats, and supraventricular premature beats, typically presenting subtle variations in atrial activation and timing irregularities. Category V includes premature ventricular contractions and ventricular escape beats, which exhibit prominent morphological deviations in the QRS complex. Category F represents fusion beats formed by the overlap of normal and ventricular depolarization. Category Q contains unclassified or unknown beats that cannot be reliably assigned to the other groups due to distortion, noise, or atypical morphology.

### 2.2. Data Preprocessing

A standardized pipeline was designed to improve signal quality and produce inputs suitable for robust heartbeat classification. The procedure included denoising, downsampling, segmentation, normalization, and class balancing.

First, baseline wander and high frequency noise were attenuated while preserving key morphological characteristics such as P waves, QRS complexes, and T waves. The signals were then downsampled from 360 Hz to 125 Hz to reduce computational cost without sacrificing diagnostically relevant information [30]. This step retained essential temporal and morphological content while producing a compact representation for deep models.

Heartbeat segmentation was performed using the annotated R peaks from the database. For each R peak, a fixed length window of 188 samples was extracted, consisting of 90 samples before the R wave and 98 samples after it. This window covered the complete P–QRS–T complex of a cardiac cycle. To mitigate inter subject amplitude variability, each segmented heartbeat underwent z score normalization [31]:

(1) $$x_{\mathrm{norm}\;}=\frac{x-\mu }{\sigma +\varepsilon }$$

where *x* represents the signal segment, μ and σ are its mean and standard deviation, respectively, and $\varepsilon =10^{-6}$ is a small constant added to prevent division by zero. This normalization procedure standardized the dynamic range of the signals, removing amplitude offset across subjects. Besides, it also ensures that signals with different magnitudes do not bias the model, stabilizes gradient updates, and improves training convergence.

Finally, to address the significant class imbalance present in the MIT-BIH dataset, the Synthetic Minority Over-sampling Technique (SMOTE) was employed [32]. Instead of simple replication on temporal waveform sequences, SMOTE generates synthetic samples for minority classes by interpolating between existing samples in the feature space. This approach effectively enhances data diversity and prevents overfitting caused by repeated instances. The resampling process was applied only to the training set to preserve the integrity of the test data and to ensure an unbiased performance evaluation.

### 2.3. ECG Signal Classification with HCTG-Net

The proposed HCTG-Net integrates a CNN branch and a Transformer branch, followed by a gated fusion module that adaptively combines the two representations. This design allows the model to jointly capture local morphological features and long-range dependencies in ECG signals, which are both crucial for robust arrhythmia detection.

**Figure 2:**
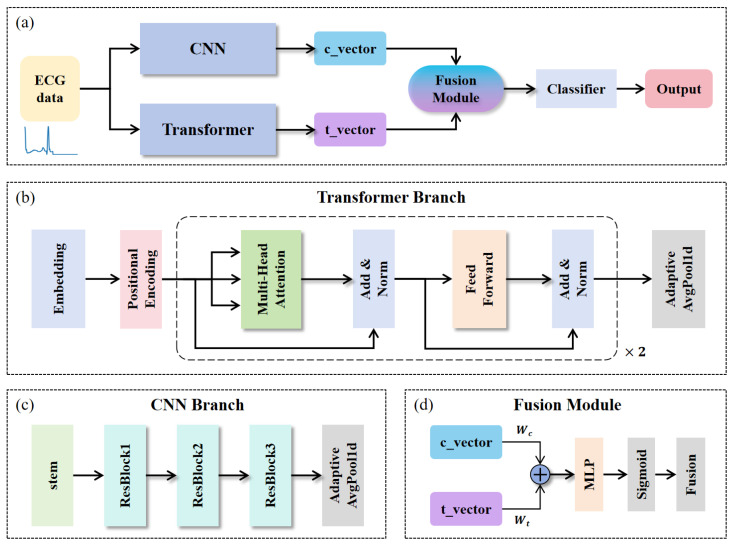
(**a**) Overall network structure of HCTG-Net. (**b**) Transformer branch. (**c**) CNN branch. (**d**) Fusion module.

Figure 2 summarizes the architecture. Specifically, Figure 2a presents the overall network structure of HCTG-Net, comprising the CNN branch, the Transformer branch, and the gated fusion module. Figure 2b details the Transformer branch, which models long-range temporal dependencies. Figure 2c shows the CNN branch, which emphasizes local morphological patterns. Figure 2d depicts the gated fusion module, which dynamically balances and integrates the local and global representations.

The CNN branch is designed to emphasize local waveform features such as QRS complexes and P/T-wave variations [33]. It begins with a stem convolutional layer consisting of 64 filters of size 7, followed by three residual blocks. Each residual block contains two convolutional layers with batch normalization and ReLU activation. Skip connections are included to stabilize gradient propagation and mitigate the vanishing gradient problem. The operation of a residual block can be expressed as:

(2) $$y=\mathrm{ReLU}\left(F\left(x\right)+W_{\mathrm{down}\;}x\right)$$

where *x* is the block input, F(·) denotes the stacked convolutional transformations, and $W_{\mathrm{down}}$ is either an identity mapping or a 1 × 1 convolution depending on dimensional alignment. The first block maintains the feature resolution with 64 channels, the second block halves the temporal resolution while increasing the depth to 128, and the third block expands the channel depth to 256. Finally, an adaptive average pooling layer produces a fixed-length representation $c\in R^{256}$.

The Transformer branch focuses on learning global dependencies across the entire heartbeat sequence [34]. Each signal segment is first projected into an embedding space of dimension $d_{\mathrm{model}}=128$. Since Transformers do not have an inherent notion of temporal order, sinusoidal positional encoding is added to the embedded sequence to explicitly represent the time position of each sample within the 188-point heartbeat window.

Equation (3) defines this sinusoidal positional encoding:

(3) $${\mathrm{PE}}_{\left(\mathrm{pos},2i\right)}=\sin\left(\frac{\mathrm{pos}\;}{10,000^{2i/d_{\mathrm{model}\;}}}\right),\;{\mathrm{PE}}_{\left(\mathrm{pos},2i+1\right)}=\cos\left(\frac{\mathrm{pos}\;}{10,000^{2i/d_{\mathrm{model}\;}}}\right)$$

The embedded sequence is processed by two stacked Transformer blocks, each comprising a multi-head self-attention (MHSA) mechanism, a position-wise feed-forward network (FFN), and residual connections with layer normalization. The MHSA mechanism is defined as:

(4) $$\mathrm{Attention}\left(Q,K,V\right)=\mathrm{softmax}\left(\frac{QK^{T}}{\sqrt{d_{k}}}\right)V$$

where queries Q, keys K, and values V are derived from linear projections of the input sequence. This mechanism enables the model to jointly capture relationships between distant signal positions. After Transformer processing, an adaptive average pooling layer aggregates the temporal information into a fixed-length vector $t\in R^{128}$.

To integrate the complementary CNN and Transformer features, both vectors are projected into a shared latent space of dimension H = 256:

(5) $$\tilde{c}=W_{c}c,\;\tilde{t}=W_{t}t$$

A gating network then generates an adaptive weight vector [35]:

(6) $$g=\sigma (\mathrm{MLP}\left(\left[\tilde{c};\tilde{t}\right]\right))$$

where σ denotes the sigmoid function and [·;·] represents vector concatenation. The MLP projects the concatenated features from dimension 2H to H through two fully connected layers. The fused representation is computed as:

(7) $$f=g⊙\tilde{c}+\left(1-g\right)⊙\tilde{t}$$

This mechanism allows the network to dynamically emphasize either local morphology or global temporal context depending on the characteristics of the input signal.

The fused feature vector is passed through a fully connected layer with 128 hidden units, followed by ReLU activation and dropout regularization [36]. The final layer applies a softmax function to obtain class probabilities for the five arrhythmia categories:

(8) $$p_{k}=\frac{\exp(z_{k})}{\sum_{j=1}^{5}\exp\left(z_{j}\right)},\;\hat{y}=\arg\max_{k}p_{k}$$

where $z_{k}$ represents the logit produced by the final linear layer for class *k*, and $p_{k}$ denotes the corresponding normalized probability. Equation (8) formalizes the probabilistic decision-making stage of HCTG-Net. The softmax operator transforms the model’s learned representation into a probability distribution across the five heartbeat categories. This process provides several important signal-interpretation benefits. First, it enables multi-class classification by assigning each heartbeat to the class with the highest probability. Second, the probability values reflect the model’s confidence in its decision, allowing uncertainty-aware interpretation of ambiguous or noisy ECG beats. Third, the softmax formulation is compatible with cross-entropy loss, which ensures stable and efficient training of the network.

The configuration of HCTG-Net is summarized in Table 1. While the table summarizes the structural configuration of each module in HCTG-Net, it is also important to highlight the specific role and contribution of these modules to ECG arrhythmia classification. The CNN branch is responsible for extracting local morphological features such as P-wave shape, QRS duration, and T-wave polarity, which are crucial for distinguishing normal, ventricular, and supraventricular beats. The use of residual blocks further stabilizes deep feature learning and preserves diagnostic waveform characteristics. The Transformer branch complements this by modeling long-range temporal dependencies within each heartbeat, enabling the network to capture contextual relationships and subtle conduction abnormalities that may not be identifiable from local morphology alone. The gated fusion module represents the key novelty of HCTG-Net: it adaptively balances and integrates the CNN-derived morphological features with the Transformer-derived global context on a per-dimension basis. This dynamic weighting mechanism allows the model to emphasize morphology-dominant cues for ventricular beats and context-dominant cues for supraventricular patterns, leading to more discriminative fused representations.

In summary, HCTG-Net combines a CNN branch for precise morphological analysis, a Transformer branch for comprehensive temporal modeling, and a gated fusion mechanism for adaptive feature integration. This hybrid design yields a unified representation that is highly effective for arrhythmia classification.

## 3. Results

### 3.1. Experimental Setup

All experiments were conducted on a personal computer equipped with an NVIDIA RTX 3080Ti GPU, an Intel Core i7 processor, and 64 GB of RAM. The implementation was carried out in Python 3.10 using the PyTorch 2.6.0 deep learning framework [37]. Training was performed with the Adam optimizer, an initial learning rate of $1\times 10^{-3}$, and weight decay of $1\times 10^{-4}$. A cosine annealing learning rate scheduler with $T_{max}=100$ epochs and a minimum learning rate of $1\times 10^{-6}$ was employed to improve convergence. The batch size was set to 256, and gradient clipping was applied with a maximum norm of 1.0 to stabilize training [38].

After constructing the complete set of segmented heartbeat samples, the resulting beat-level dataset was randomly divided into training, validation, and testing subsets. For model development, 80% of the training data was used for training, while the remaining 20% served as the validation set for hyperparameter tuning and early stopping. The final test set was held out and used exclusively for the evaluation of model performance.

### 3.2. Evaluation Metrics

The classification performance of HCTG-Net was evaluated using multiple quantitative metrics to ensure a comprehensive assessment. The selected metrics included accuracy, precision, recall, and the F1-score [39,40]. These metrics together provided a balanced view of both overall and class-wise performance.

Accuracy measured the overall proportion of correctly classified samples and was computed as:

(9) $$\;\mathrm{Accuracy}\;=\frac{\mathrm{TP}+\mathrm{TN}}{\mathrm{TP}+\mathrm{TN}+\mathrm{FP}+\mathrm{FN}}$$

where TP, TN, FP, and FN denote true positives, true negatives, false positives, and false negatives, respectively.

Precision quantified the proportion of correctly predicted positive samples among all predicted positives:

(10) $$\;\mathrm{Precision}\;=\frac{\mathrm{TP}}{\mathrm{TP}+\mathrm{FP}}$$

Recall represented the proportion of actual positive samples correctly identified by the model:

(11) $$\;\mathrm{Recall}\;=\frac{\mathrm{TP}}{\mathrm{TP}+\mathrm{FN}}$$

The F1-score was calculated as the harmonic mean of precision and recall, providing a single measure that balances both sensitivity and precision:

(12) $$F1=2\cdot \frac{\;\mathrm{Precision}\;\cdot \;\mathrm{Recall}\;}{\;\mathrm{Precision}\;+\;\mathrm{Recall}\;}$$

To clearly highlight the characteristics and practical differences among the four evaluation metrics used in this study, we provide a comparative summary in Table 2.

The combination of accuracy, precision, recall, and F1-score offered a comprehensive evaluation of the model’s performance. Accuracy reflected the overall classification capability, while precision and recall highlighted the model’s sensitivity to positive predictions. The F1-score captured the trade-off between these two measures, ensuring a fair and reliable assessment of arrhythmia classification performance on the MIT-BIH dataset.

### 3.3. Overall Performance of HCTG-Net

**Figure 3:**
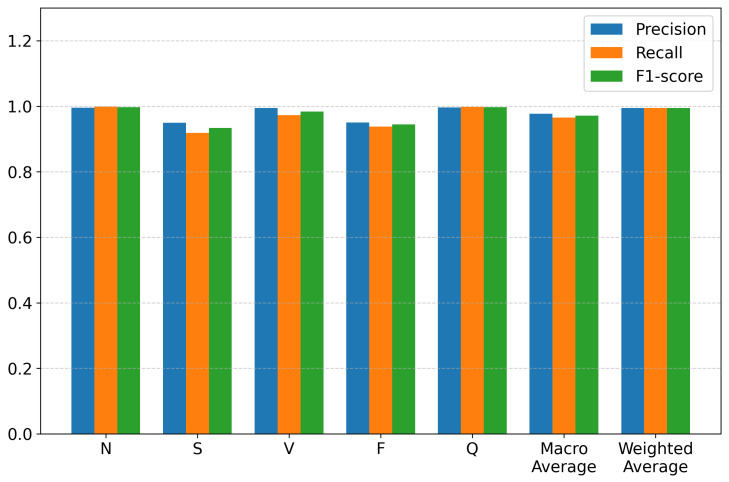
Classification performance of HCTG-Net across heartbeat categories.

The proposed HCTG-Net was comprehensively evaluated on the MIT-BIH Arrhythmia Database according to the AAMI heartbeat classification standard. Table 3 summarizes the classification results across the five major heartbeat categories, reporting precision, recall, and F1-score for each class, together with the overall average metrics. The detailed performance distribution is also illustrated in Figure 3.

The results in Table 3 demonstrate that HCTG-Net achieves consistently high performance across all heartbeat classes. The N and Q classes exhibit near-perfect precision, recall, and F1-score, indicating that the model is highly effective at recognizing both common and miscellaneous waveform patterns. The V class also shows excellent performance, with an F1-score of 0.9839. This result highlights the model’s ability to capture the distinctive morphological characteristics of ventricular arrhythmias, which are clinically critical to detect accurately.

Performance for the S and F categories is slightly lower, with F1-scores of 0.9338 and 0.9441, respectively. These reductions are expected due to the inherent class imbalance in the MIT-BIH dataset and the subtle morphological similarities between these arrhythmias and normal beats. The S class in particular is known to exhibit diverse and less pronounced waveform deviations, making it a challenging category for automated classification. Nevertheless, HCTG-Net still maintains strong performance for these minority classes, demonstrating its ability to generalize across both dominant and less frequent arrhythmia types.

Aggregated results further confirm the strong classification capability of the proposed model. The macro-average precision of 0.9774 and macro-average F1-score of 0.9711 reflect balanced performance across all classes, independent of class frequency. The weighted averages, which account for class distribution, are even higher due to the excellent performance on dominant categories. The overall accuracy of 0.9946 demonstrates that the hybrid CNN–Transformer architecture and gated fusion module effectively capture both local morphological details and long-range temporal dependencies in ECG signals.

**Figure 4:**
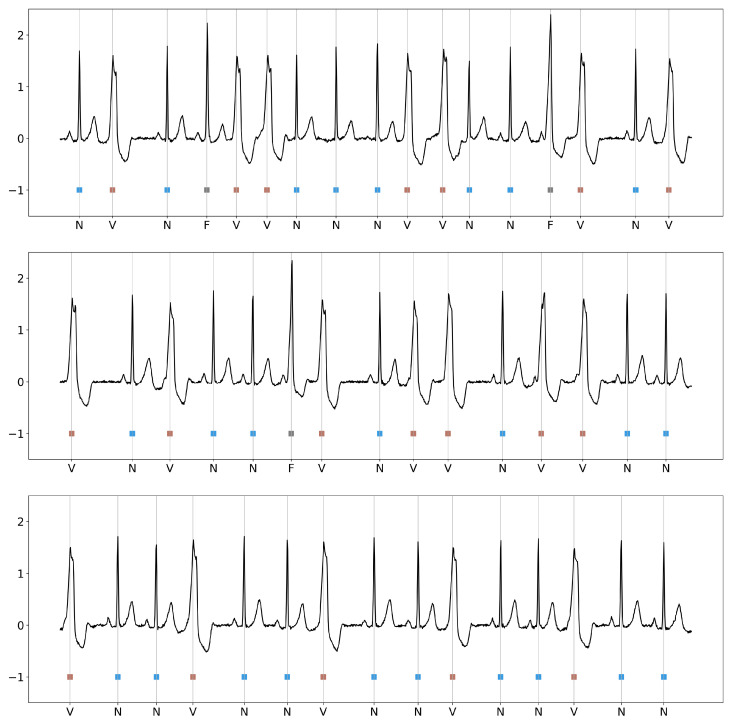
Three representative 10-s segments from the original MIT-BIH Arrhythmia Database recordings.

A representative example of the input data analyzed by HCTG-Net is shown in Figure 4, which displays three 10-s segments of the original MIT-BIH ECG recording. Each heartbeat in the segment is annotated with its corresponding AAMI class label. This visualization highlights the morphological variability among beats.

**Figure 5:**
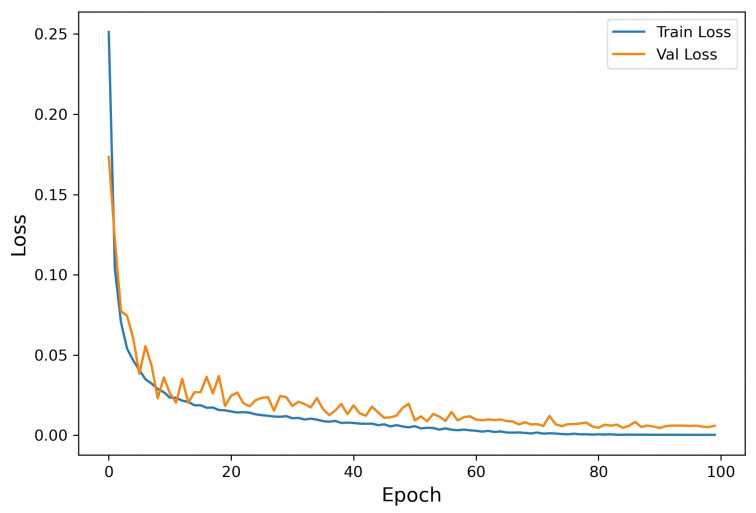
Training and validation loss curves of HCTG-Net during model optimization.

To further analyze the training stability and convergence behavior of HCTG-Net, the training and validation loss curves across epochs are illustrated in Figure 5. As shown, the training loss decreases rapidly during the initial phase and gradually plateaus after convergence, indicating effective learning and stable optimization. The smooth convergence trend demonstrates that proposed HCTG-Net enables efficient gradient propagation and stable feature learning during training.

In addition to accuracy and convergence performance, computational efficiency was also evaluated to assess the practicality of HCTG-Net in real-time clinical applications. During inference, the model processed 10,000 heartbeat samples in 1.507 s, corresponding to an average reporting time of approximately 0.15 ms per sample. This remarkably low latency demonstrates that HCTG-Net is capable of near real-time arrhythmia classification, making it suitable for continuous ECG monitoring scenarios such as Holter analysis, bedside cardiac surveillance, and wearable health devices.

### 3.4. Confusion Matrix Analysis

**Figure 6:**
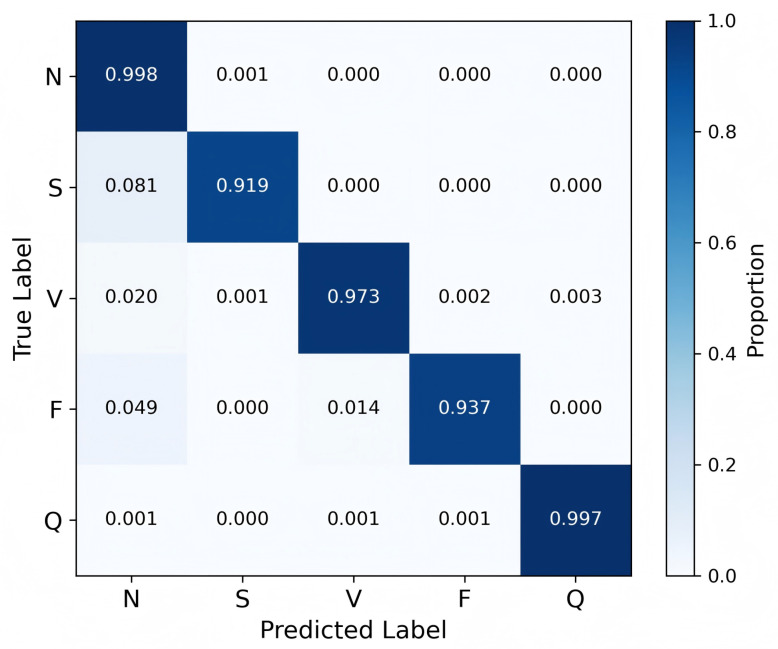
Confusion matrix of HCTG-Net on the MIT-BIH test set.

To further evaluate the classification capability of HCTG-Net, a confusion matrix was generated on the MIT-BIH test set, as shown in Figure 6. The confusion matrix provides a detailed visualization of the model’s ability to accurately identify each heartbeat type and the nature of the misclassification patterns.

The confusion matrix shows that the majority class N achieved the highest recognition performance, with the vast majority of samples correctly classified. Only a very small number of N beats were misclassified as S, V, F, or Q, indicating that the model effectively captures the typical P–QRS–T morphology and rhythm characteristics of normal ECG patterns. The strong diagonal dominance for this class confirms its clear separability and reflects the abundance of normal beats available during training.

The Q class shows a recall of 0.997, with minor confusion distributed across N, V, and F. Because this category includes a heterogeneous set of beats that do not fit into other classes, occasional overlap is consistent with its diverse waveform characteristics.

The V class also exhibits excellent separability. This result suggests that the CNN branch of HCTG-Net successfully extracts discriminative morphological features, such as widened QRS complexes or abnormal ventricular depolarization patterns, which are characteristic of V beats. The high recall for this class is clinically important because ventricular arrhythmias often carry greater diagnostic risk [41].

The F obtains a recall of 0.937, with limited confusion primarily involving N and V. Since fusion beats combine features of both normal and ventricular depolarizations, slight ambiguity is unavoidable. The model’s ability to classify most fusion beats correctly demonstrates that the gated fusion mechanism effectively integrates local and global features to distinguish their hybrid morphology. The S class achieved a recall of 0.919, with occasional misclassification as N, likely due to morphological similarities between supraventricular and normal beats.

Overall, the confusion matrix confirms the robustness of HCTG-Net in accurately distinguishing both majority and minority heartbeat categories.

While the confusion matrix demonstrates the overall robustness of HCTG-Net, it is also essential to consider the clinical implications of its residual misclassifications. Compared with conventional handcrafted feature-based methods [42,43,44], which rely on fixed morphological descriptors, the proposed model reduces the likelihood of clinically critical errors, such as misclassifying V for N. These high-risk confusions are particularly concerning, as they may lead to missed diagnoses of ventricular arrhythmias. The deep hierarchical representations learned by HCTG-Net effectively suppress such errors, highlighting its enhanced sensitivity to subtle morphological and temporal variations in ECG waveforms.

Furthermore, the architecture of HCTG-Net inherently supports probabilistic confidence estimation for each prediction, providing a practical mechanism for clinical decision support. Predictions with low confidence can be automatically flagged as uncertain cases, prompting cardiologists to conduct additional diagnostic evaluations—such as repeat Holter monitoring, exercise ECG, dobutamine stress ECG, or a short 12-lead ECG—to confirm the diagnosis. This feedback mechanism transforms the model from a passive classifier into an active assistant, thereby enhancing diagnostic reliability and helping clinicians prioritize cases that require closer examination. Integrating model confidence into the clinical workflow could further improve patient safety and increase the trustworthiness of AI-assisted arrhythmia screening in real-world applications.

### 3.5. Visualization of Feature Representation

To gain deeper insights into the feature learning capability of HCTG-Net, the high-dimensional representations from the final fused feature space were visualized using t-distributed Stochastic Neighbor Embedding (t-SNE) [45]. This nonlinear dimensionality reduction technique projects the learned 256-dimensional embeddings onto a two-dimensional space while preserving local neighborhood relationships, thereby illustrating the clustering behavior of different heartbeat classes.

**Figure 7:**
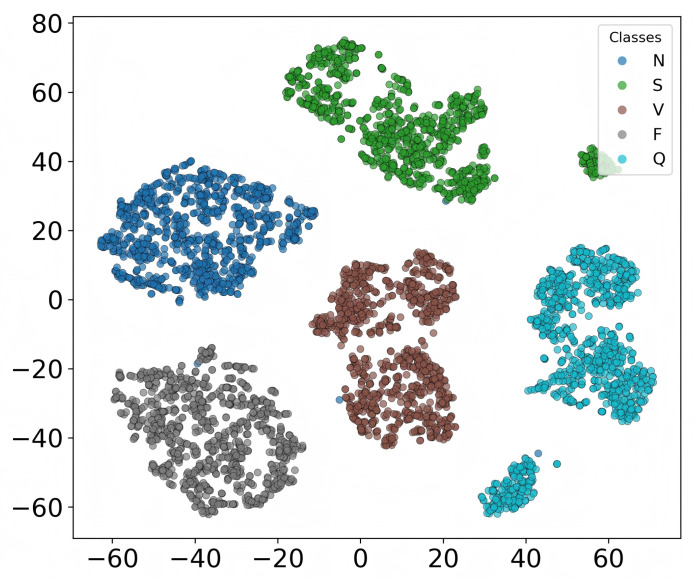
t-SNE visualization of the learned representation on the MIT-BIH test set.

As shown in Figure 7, samples belonging to the same category form compact clusters, with distinct boundaries observed between most classes. Normal beats N and fusion beats F form clear and well-isolated clusters, indicating that HCTG-Net effectively captures their discriminative morphological characteristics. Supraventricular arrhythmias S and the Q category exhibit more scattered distributions, reflecting the greater intra-class variability and morphological diversity of these beats. A small degree of overlap is observed between V and Q, suggesting partial similarity in certain waveform patterns or temporal features.

These findings confirm that HCTG-Net successfully learns discriminative and semantically meaningful feature representations by leveraging the complementary strengths of its CNN and Transformer branches.

### 3.6. Comparative Study with Previous Methods

To further validate the effectiveness of the proposed HCTG-Net, we compared its performance with several existing methods previously reported in the literature on the MIT-BIH Arrhythmia Database. The compared studies are those of Curtin et al. [44], Wang et al. [46], Sharma et al. [47], Kumar et al. [48], Zhuang et al. [49], Wu et al. [50], Irsyad et al. [51], Tchinda et al. [52], Choudhury et al. [53], and Bahrami et al. [54]. Table 4 summarizes the comparative results in terms of accuracy, precision, recall, and F1-score.

From the results in Table 4, it is evident that the proposed HCTG-Net achieves the highest overall accuracy (0.9946) and F1-score (0.9711) among all competing methods. Although its precision (0.9774) is slightly lower than that of Kumar et al., Zhuang et al. and Sharma et al., HCTG-Net demonstrates superior balance across all metrics, achieving both high recall and excellent generalization capability. This balance indicates that the model not only identifies arrhythmias accurately but also maintains strong robustness against class imbalance and morphological variability in ECG waveforms.

It is also worth noting that the earlier study by Curtin et al. [44], which represents a typical handcrafted feature-based approach, achieved an overall accuracy of 0.9430 and F1-score of 0.9653. Such methods rely heavily on manually designed morphological descriptors, statistical parameters, and expert-defined thresholds. While these approaches are effective in capturing certain QRS complex characteristics, they are inherently limited in modeling complex waveform morphologies and long-term temporal dependencies. In contrast, HCTG-Net leverages deep hierarchical feature extraction and gated fusion between CNN and Transformer branches, enabling it to learn both local morphological and global temporal patterns directly from raw ECG data without manual feature engineering. This capability allows HCTG-Net to achieve markedly higher accuracy and robustness, confirming its superiority over traditional hand-crafted algorithms in handling diverse and noisy ECG signals.

### 3.7. Ablation Study

In this section, an ablation study is carried out to investigate the influence of the major architectural components of the proposed HCTG-Net on arrhythmia classification performance. The analysis focuses on three core modules: the CNN branch, responsible for extracting local morphological features from ECG signals; the Transformer branch, which captures long-range temporal dependencies across heartbeats; and the gated fusion mechanism, which adaptively integrates features from both branches. To systematically examine the contribution of each component, several simplified variants of HCTG-Net were evaluated. These include a CNN-only version that focuses solely on local feature learning, a Transformer-only version emphasizing global temporal context, a hybrid CNN–Transformer model without the gating mechanism, and the full proposed HCTG-Net with all components enabled. All experiments were conducted on the MIT-BIH Arrhythmia Database under identical preprocessing, training, and testing conditions. The results of the ablation analysis are summarized in Table 5.

From Table 5, it can be observed that the CNN-only model is superior to the Transformer-only model in terms of accuracy but falls short in recall, demonstrating that convolutional layers are adept at capturing localized ECG patterns such as QRS complexes but are less effective in modeling the temporal dependencies required for detecting complex arrhythmias. The Transformer-only configuration shows an improvement in recall, indicating its capability to model long-term dependencies, though it slightly sacrifices precision due to the absence of spatially localized information.

When both the CNN and Transformer branches are combined without the gated fusion mechanism, performance improves significantly, verifying that local and global features are complementary in ECG classification. However, without adaptive weighting, the feature combination may be redundant or uneven, leading to suboptimal learning of discriminative representations.

The full HCTG-Net, which integrates CNN, Transformer, and the gated fusion mechanism, achieves the highest overall accuracy and F1-score. The learnable gating structure dynamically adjusts the contribution of each branch according to the input characteristics, allowing the model to emphasize local features during sharp waveform transitions and global dependencies when temporal variations dominate. This adaptability ensures balanced performance across all metrics and enhances model robustness.

## 4. Discussion

The results obtained on the MIT-BIH Arrhythmia Database demonstrate that the proposed HCTG-Net achieves both high overall performance and balanced behavior across all heartbeat categories. As summarized in Table 4, HCTG-Net attains the highest accuracy, recall, and F1-score among all compared methods. This suggests that the model is not only capable of correctly classifying the dominant N beats, but is also effective at detecting less frequent yet clinically important arrhythmias such as S and F beats. In addition, the very low inference latency indicates that the method is suitable for real-time monitoring scenarios, which is essential for practical deployment in ambulatory and bedside environments.

From a modeling perspective, the main strength of HCTG-Net lies in its ability to integrate complementary local and global information within a single unified architecture. The CNN branch specializes in extracting fine-grained morphological features such as QRS width, P-wave and T-wave shapes, and subtle repolarization changes. These local patterns are critical for distinguishing, for example, ventricular beats from normal beats, where large and sharp changes in waveform morphology are present. In parallel, the Transformer branch focuses on modeling long-range temporal dependencies and rhythm context, enabling the network to recognize arrhythmias that manifest primarily through timing irregularities or more subtle morphological variations over time. By learning these two types of information independently and in parallel, HCTG-Net constructs a richer representation than methods that rely solely on CNNs or solely on sequence models.

A central novelty of the proposed network is the learnable gated fusion mechanism that combines the outputs of the CNN and Transformer branches. Instead of concatenating features or applying a fixed fusion rule, HCTG-Net employs a gating network that produces per-dimension importance weights for morphology-dominated versus context-dominated information. This design allows the model to dynamically emphasize the most informative branch for each feature dimension and for each input heartbeat. For example, the gate can favor CNN-derived features when the QRS complex exhibits significant distortion, as is typical in ventricular arrhythmias, and shift toward Transformer-derived features when discriminative information lies in the temporal pattern of successive beats, as in many supraventricular arrhythmias. The ablation study confirms that this adaptive fusion yields substantial gains over simple combinations of CNN and Transformer branches, indicating that performance improvements are driven by more effective feature integration rather than merely increased model capacity.

Several additional design choices contribute to the high accuracy and F1-score achieved by HCTG-Net. First, the use of residual connections in the CNN branch helps preserve the continuity of clinically relevant waveform patterns across layers, reducing the risk that subtle morphological details will be attenuated by deep transformations. Second, both branches are projected into a shared latent space before fusion, which improves feature-scale alignment and stabilizes optimization. Third, the preprocessing pipeline, which comprises denoising, heartbeat segmentation, and z-score normalization, reduces inter-subject amplitude variability and improves temporal alignment of P–QRS–T complexes. These steps reduce intra-class variance and facilitate more discriminative feature learning. Finally, the use of SMOTE and class-balancing strategies during training mitigates the effect of class imbalance, and directly improves performance metrics.

CNNs were popularized in biomedical signal processing for handling very large and high-dimensional data such as EEG and invasive electrophysiological recordings. Despite the comparatively smaller data volume per ECG record, they remain highly effective for ECG analysis. ECG waveforms exhibit strong local structure: the P wave, QRS complex, and T wave occur within compact temporal windows and carry most of the diagnostic information. One-dimensional CNNs are well matched to this structure, as they can efficiently learn filters that detect these characteristic motifs, their relative timings, and subtle shape variations. The ablation experiment shows that even a CNN-only variant of HCTG-Net achieves a high accuracy of 0.9697, underscoring the effectiveness of convolutional feature extraction for ECG. Within the full HCTG-Net, the CNN branch thus serves as a robust backbone for morphology-aware representation learning, while the Transformer branch and gated fusion further refine these features with temporal context and adaptive weighting.

Despite these strengths, several limitations of the proposed HCTG-Net should be acknowledged. First, the incorporation of Transformer blocks and a gated fusion network increases architectural complexity and computational overhead compared to lightweight CNN-only models. Although the current implementation achieves low inference latency on modern GPU hardware, deployment in extremely resource-constrained embedded systems may require further model compression, pruning, or quantization. Second, the relatively high model capacity increases the risk of overfitting, especially when applied to smaller or less diverse datasets. This risk is partly mitigated by the use of regularization techniques and data-balancing strategies, but careful tuning of training protocols remains necessary when adapting HCTG-Net to new datasets. Third, like many deep learning models, HCTG-Net remains a black-box predictor from the perspective of clinicians. So additional interpretability tools would be valuable to enhance trust and facilitate clinical adoption.

## 5. Conclusions

In this study, we proposed HCTG-Net, a novel Hybrid CNN–Transformer Network with Gated Fusion for automatic arrhythmia detection from ECG signals. The model was designed to integrate the complementary advantages of convolutional and self-attention mechanisms within a unified architecture. Specifically, the CNN branch effectively captures localized morphological features such as QRS complex and P–T wave patterns, while the Transformer branch models long-range temporal dependencies and contextual relationships across consecutive heartbeats. The proposed gated fusion mechanism adaptively balances the contribution of these two branches, allowing the network to dynamically emphasize either local or global information depending on the signal characteristics.

Extensive experiments conducted on the MIT-BIH Arrhythmia Database demonstrated that HCTG-Net achieves superior performance compared with existing methods in terms of accuracy, recall, and F1-score. The visualization of the learned feature space further confirmed that the model generates compact and well-separated clusters for different heartbeat categories, indicating strong discriminative feature learning. Ablation studies validated the importance of each module—CNN, Transformer, and gated fusion—revealing that their synergistic combination leads to substantial gains in both classification accuracy and model robustness.

Overall, the proposed HCTG-Net provides an efficient, accurate, and interpretable framework for ECG-based arrhythmia detection. Its hybrid and adaptive design enhances the generalization ability across diverse cardiac patterns and can be readily extended to other biomedical signal analysis tasks. Future work will focus on optimizing the model for real-time deployment in wearable and mobile healthcare systems, integrating multi-lead ECG data, and exploring domain adaptation techniques to improve cross-dataset robustness.

## Figures and Tables

**Table 1:** Key architectural components of HCTG-Net.

Module	Layer/Operation	Configuration (Channels/Size/Parameters)	Output Dimension
CNN Branch	Stem Conv + BN + ReLU	Conv1D (1→64), kernel = 7, stride = 1, padding = 3	[B, 64, L]
	Residual Block 1	2×Conv1D (64→64), kernel = 3, stride = 1	[B, 64, L]
	Residual Block 2	2×Conv1D (64→128), kernel = 3, stride = 2	[B, 128, L/2]
	Residual Block 3	2×Conv1D (128→256), kernel = 3, stride = 1	[B, 256, L/2]
	Adaptive AvgPooling	Pool over time dimension	[B, 256]
Transformer Branch	Linear Embedding	Input [B, L, 1] → [B, L, 128]	[B, L, 128]
	Positional Encoding	Sinusoidal, *d* = 128	[B, L, 128]
	Transformer Layer × 2	MHSA (nhead = 4), FFN = 256, dropout = 0.1	[B, L, 128]
	Adaptive AvgPooling	Pool over sequence length	[B, 128]
Fusion Module	Projection Layers	CNN: 256→256, Transformer: 128→256	[B, 256]
	Gating Network	MLP(512→256→256), sigmoid	[B, 256]
	Fusion	$f\;=\;g⊙\tilde{c}+\left(1-g\right)⊙\tilde{t}$	[B, 256]
Classifier	Fully Connected + ReLU + Dropout	FC(256→128), dropout = 0.3	[B, 128]
	Output Layer	FC(128→5), Softmax	[B, 5]

CNN: Convolutional Neural Network; MHSA: Multi-Head Self-Attention.

**Table 2:** Comparison of the four evaluation metrics used in this study.

Metric	Definition/Purpose	Strengths and Limitations
Accuracy	Proportion of all samples that are correctly classified.	Provides an overall performance measure, but can be misleading when classes are imbalanced.
Precision	Probability that a predicted positive sample truly belongs to the positive class.	Effective for reducing false alarms, although it does not account for missed detections.
Recall	Proportion of actual positive samples that are correctly identified.	Particularly important in medical diagnosis to avoid missed arrhythmias, but may increase false positives.
F1-score	Harmonic mean of precision and recall, balancing both types of errors.	More robust under class imbalance, though its interpretation can be less intuitive in multiclass settings.

**Table 3:** Classification performance metrics for each heartbeat class.

Class	Precision	Recall	F1-Score
N	0.9958	0.9984	0.9971
S	0.9495	0.9186	0.9338
V	0.9948	0.9732	0.9839
F	0.9507	0.9375	0.9441
Q	0.9962	0.9975	0.9968
Macro Average	0.9774	0.9650	0.9711
Weighted Average	0.9945	0.9946	0.9945
Accuracy	-	-	0.9946

**Table 4:** Comparison of HCTG-Net with previous methods on the MIT-BIH Arrhythmia Database.

Methods	Accuracy	Precision	Recall	F1-Score
Curtin et al.	0.9430	0.9730	0.9600	0.9653
Wang et al.	0.9340	0.9732	0.8983	0.9344
Sharma et al.	0.9853	0.9825	0.9568	0.9695
Kumar et al.	0.9866	0.9892	0.9388	0.9634
Zhuang et al.	0.9702	0.9844	0.9474	0.9652
Wu et al.	0.9570	0.7778	0.8810	0.8260
Irsyad et al.	0.9829	0.9375	0.8760	0.9037
Tchinda et al.	0.9842	0.9523	0.8760	0.9126
Choudhury et al.	0.9385	0.9422	0.9342	0.9383
Bahrami et al.	0.9913	0.9504	0.9228	0.9364
Proposed HCTG-Net	0.9946	0.9774	0.9650	0.9711

**Table 5:** Ablation study results of different model variants.

Model Variant	Accuracy	Precision	Recall	F1-Score
CNN-only	0.9697	0.8399	0.9181	0.8741
Transformer-only	0.9629	0.7986	0.9262	0.8512
CNN + Transformer (without gated fusion)	0.9825	0.9168	0.9072	0.9116
Proposed HCTG-Net	0.9946	0.9774	0.9650	0.9711

## Data Availability

The original data and codes of this work can be requested from the corresponding author.
